# Risk factors for diabetic ketoacidosis in acute pancreatitis patients with type 2 diabetes

**DOI:** 10.1186/s12876-023-02869-2

**Published:** 2023-07-27

**Authors:** Lin Li, Linzhen Li

**Affiliations:** Departments of Gastroenterology, First Affiliated Hospital of Wannan Medical College, 241001 Wuhu, Anhui Province P. R. China

**Keywords:** Acute pancreatitis, Type 2 diabetes, Diabetic ketoacidosis, Risk factors

## Abstract

**Background and purpose:**

In cinical, some acute pancreatitis patients with diabetes may have diabetic ketoacidosis (DKA). However, the risk factors for DKA in these patients remain unclear. The purpose of this study is to analyze the risk factors for DKA in acute pancreatitis patients with type 2 diabetes.

**Patients and methods:**

Twenty-five patients were included in this prospective single-centre study to analyze the incidence and risk factors for DKA in acute pancreatitis patients with type 2 diabetes.

**Results:**

Seven of the twenty-five patients (28%) developed DKA within 48 h of admission. According to whether they had DKA, the twenty-five AP patients were divided into DKA group and non-DKA group. There were significant differences in age (*P* = 0.014), BMI (*P* = 0.034), poor previous blood glucose control (*P* < 0.001) and uric acid concentration (*P* = 0.041), but no statistically significant differences in sex (*P* = 0.597), number of drinkers (*P* = 0.407), number of smokers (*P* = 1.000), triglyceride level (*P* = 0.389) and total cholesterol concentration (*P* = 0.534) between the two groups. In both groups, 1 patients had severe pancreatitis, and the difference was no statistically significant (*P* = 0.490).

**Conclusions:**

The incidence of DKA in AP patients with diabetes is high. Age, BMI, worse glycemic control and uric acid concentration may be predictors of DKA in AP patients with diabetes.

**Supplementary Information:**

The online version contains supplementary material available at 10.1186/s12876-023-02869-2.

## Introduction

Acute pancreatitis (AP) refers to a series of symptoms caused by the activation of pancreatic enzymes in the pancreas, resulting in the digestion of the pancreas itself. Acute pancreatitis can be caused by various reasons, such as gallstones, hyperlipidaemia and excessive alcohol consumption [1–3]. According to the Revised Atlanta Classification (RAC), AP can be divided into mild acute pancreatitis, moderately severe acute pancreatitis and severe acute pancreatitis [4]. A diagnosis of acute pancreatitis requires two out of three criteria: (1) abdominal pain, (2) a serum amylase or lipase increased at least threefold, and (3) findings consistent with pancreatitis on cross-sectional abdominal imaging [computed tomography (CT) or magnetic resonance imaging (MRI)] [4, 5].

Diabetes mellitus (DM) is a group of disorders of carbohydrate, protein and fat metabolism caused by absolute or relative insufficiency of insulin secretion and/or dysfunction of insulin utilization. Acute complications of diabetes include ketoacidosis and hyperglycemia hyperosmolar state. Diabetic ketoacidosis (DKA) is the most common acute hyperglycaemic emergency in people with diabetes mellitus. The diagnostic criteria for DKA should meet the following: (1) the individual must have been previously diagnosed with diabetes, (2) a pH < 7.3 or a serum bicarbonate of < 15.0 mmol/l, and (3) plasma beta-hydroxybutyrate concentrations of ≥ 3.0 mmol/l, or urine ketones of more than 2+ [6, 7].

At the pathogenesis of AP, inflammatory mediators and cytokines, such as tumor necrosis factor (TNF), play an important role. Animal studies have shown that blocking the TNF can minimize pancreatic damage [8, 9]. Hyperglycemia has been shown to induce proinflammatory cytokines in monocyctic cells [10]. Certain cytokines, such as TNF, impair insulin action in peripheral tissue [11]. Diabetes mellitus and its complications are characterized with high burden of inflammation. Is DKA more likely to occur in AP patients with diabetes mellitus?

As is known to all, insulin is a hormone secreted by cells in the pancreas that regulates blood sugar levels. When acute pancreatitis occurs, it may result in insufficient insulin production, which can affect blood sugar levels. In cinical, some patients with acute pancreatitis and diabetes mellitus may have DKA. Risk factors for DKA in patients with acute pancreatitis and diabetes mellitus have not been studied. Therefore, the purpose of this prospective study was to investigate the risk factors for DKA in patients with acute pancreatitis and diabetes mellitus.

## Methods

### Ethical considerations

The research was performed according to the Declaration of Helsinki including patients’ consent. The study was approved by the local Ethics Committee.

### Patients and study design

A total of 25 patients were included in this prospective single-centre study (July 2020-December2022). The inclusion criteria were as follows: (1) a patient with acute pancreatitis who had previously been diagnosed with diabetes, (2) come to our department within 24 h of onset, (3) Blood glucose levels were measured by nurse every 4 h during hospitalization. The exclusion criteria were as follows: (1) patients refused to monitor blood glucose levels, (2) discharge or death within 48 h of hospitalization. Gender, age, body mass index(BMI), alcohol drinking, smoking were recorded in the 25 patients when they were first admitted to hospital. On the second day of hospitalization, blood was drawn to check triglyceride level, total cholesterol concentration and uric acid concentration, recording these three values as well. After admission, all the 25 patients began to monitor their blood glucose by nurse every 4 h to observe whether DKA occurred within 48 h after admission ( If blood glucose levels exceeds 15mmol/L, insulin is used for hypoglycemic treatment ). The diagnostic criteria for DKA should meet the following: (1) the individual must have been previously diagnosed with diabetes, (2) a pH < 7.3 or a serum bicarbonate of < 15.0 mmol/l, and (3) plasma beta-hydroxybutyrate concentrations of 3.0 mmol/l,or urine ketones of more than 2+ [6, 7]. The 25 patients were divided into two groups according to whether they had DKA, and the differences between the two groups were compared for gender, age, BMI, number of drinkers, number of smokers, triglyceride level, total cholesterol concentration and uric acid concentration.

### Statistical analysis

Descriptive data are expressed in terms of median (interquartile) or percentage. All numerical variables were tested for normal distribution. Mann-whitney U test was used for nonparametric tests, and Chi-square test or Fisher’s exact test was used for categorical variables. SPSS 21.0 software was used for statistical analysis.A *P*-value < 0.05 indicated statistical significance.

### Results

#### Basic clinical characteristics

A total of 25 patients were included in the study. All the 25 patients had type 2 diabetes. There were 20 males and 5 females. The median age (interquartile spacing) for all patients was 45 (26.50–60.00) years. In this study, the median (interquartile spacing) of BMI was 25.16 (23.41–27.86). Fourteen (56%) patients had a history of drinking or were still drinking. Thirteen (52%) patients had a history of smoking or were still smoking. The median (interquartile range) for triglyceride level, total cholesterol concentration and uric acid concentration were 2.02 (0.81–16.08) mmol/L, 5.14 (3.62–9.59) mmol/L and 352 (227–473) umol/L, respectively. Of the 25 patients, 2 (8%) patients were SAP. Twelve (48%) patients had poor previous blood glucose control. According to the Chinese Guidelines for Diagnosis and Treatment of diabetes, poor glycemic control was defined as fasting blood glucose > 7mmol/L or postprandial blood glucose > 10 mmol/L or HbA1c > 7%. Seven (28%) developed DKA within 48 h of admission. (Table 1).

**Table 1:** Clinical features of the patients

Sex (M/F)	20/5
Age (years):M(QR)	45 (26.50–60.00)
BMI: M(QR)	25.16 (23.41–27.86)
Number of drinkers: n(%)	14 (56%)
Number of smokers: n(%)	13 (52%)
Triglyceride level: M(QR), mmol/L	2.02 (0.81–16.08)
Total cholesterol concentration: M(QR), mmol/L	5.14 (3.62–9.59)
Uric acid concentration: M(QR), umol/L	352 (227–473)
Poor glycemic control: n(%)	12 (48%)
SAP: n(%)	2 (8%)
DKA: n(%)	7 (28%)

BMI:body mass index, SAP:severe acute pancreatitis, DKA:diabetic ketoacidosis, M:median, QR:Quartile Range

### Analysis of the differences between DKA group and non-DKA group

In this study, 25 AP patients were divided into DKA group and non-DKA group according to whether they had DKA. There were significant differences in age (*P* = 0.014), BMI (*P* = 0.034), but no statistically significant differences in sex (*P* = 0.597), number of drinkers (*P* = 0.407), number of smokers (*P* = 1.000). There was significant difference in uric acid concentration (*P* = 0.041), but no statistically significant differences in triglyceride level (*P* = 0.389) and total cholesterol concentration (*P* = 0.534) between the two groups ( Table 2). In the DKA group, all the 7 (100%) patients had poor previous blood glucose control, but in the non-DKA group, only 5 (27.78%) patients had poor previous blood glucose control. The difference was statistically significant. Only 1 patient in both groups had severe pancreatitis, and the difference was no statistically significant (P = 0.490).

**Table 2:** **A**nalysis of the differences between DKA group and non-DKA group

	DKA group, n = 7	Non-DKA group, n = 18	*P*值
Sex:female, n(%)	2(28.57%)	3(16.67%)	0.597
Age (years):M(QR)	26 (24.00–39.00)	47.5 (34.75-67.00)	0.014
BMI: M(QR)	27.4(26.00-29.75)	24.85 (22.88–26.96)	0.034
Number of drinkers: n(%)	5(71.43%)	9(50%)	0.407
Number of smokers: n(%)	4(57.14%)	9(50%)	1.000
Triglyceride level: M(QR), mmol/L	5.14 (1.94–17.57)	1.86 (0.80-16.53)	0.389
Total cholesterol concentration: M(QR), mmol/L	6.85 (4.50-10.41)	4.90 (3.46–9.48)	0.534
Uric acid concentration: M(QR), umol/L	442 (352.00-698.00)	297.5 (193.75–459.00)	0.041
Poor glycemic control: n(%)	7 (100%)	5 (27.78%)	0.002
SAP: n(%)	1 (14.29%)	1 (5.56%)	0.490

BMI:body mass index, SAP:severe acute pancreatitis, DKA:diabetic ketoacidosis, M:median, QR:Quartile Range

## Discussion

Acute pancreatitis is a common disease in gastroenterology. AP is defined as the acute inflammation of the pancreas, leading to local and systemic complications, including pancreatic edema, necrosis, hemorrhage or infection, accompanied with pancreatic pseudocyst, pancreatic abscess, and orther organs dysfunction or failure [12]. In patients with acute pancreatitis combined with hyperglycemia or preexisting diabetes, blood sugar should be actively monitored [13], to prevent the occurrence of DKA and other complications. The prevalence of DKA varies across the world. A research by Dabelea et al. in 2014 showed that among youth with type 2 diabetes (n = 1425), DKA prevalence was 11.7% in 2002–2003, and 5.7% in 2008–2010 [14]. A retrospective study by Fu Y et al. published in 2022 [15] showed that the incidence of DKA in AP patents with diabetes was 19.9%. In our study, the incidence of DKA in AP patents with type 2 diabetes was 28%, much higher than the study above. The probability of DKA in type 2 diabetes patients with AP may be higher than these without AP, so blood glucose should be closely monitored during hospitalization.

Previous studies have found that DKA is related to age, gender, alcohol, BMI, smoking, worse glycemic control [14, 16–19]. The study by Fu Y et al. [15] also analyzed the risk factors for DKA in AP patients with type 2 diabetes, and they found that DKA patients tended to be younger, more obese and had higher blood glucose and triglyceride level. But the study did not analyze whether uric acid concentration was a risk factor. In our study, there were significant differences in age (*P* = 0.014), BMI (*P* = 0.034) and worse glycemic control (*P*<0.001), consistent with previous studies. But there were no statistically significant differences in sex (P = 0.597), number of drinkers (P = 0.407), number of smokers (P = 1.000). The reason why this is inconsistent with previous studies may be due to the small sample size of this study. We also looked at whether there were statistical differences in triglyceride level, total cholesterol concentration and uric acid concentration between the two groups. We found only uric acid concentration was significantly different between the two groups.

As uric acid was an endogenous pro-inflammatory signal released from injured cells, high serum uric acid levels can trigger inflammation [20]. A retrospective cross-sectional cohort study published in 2022 [21] showed that uric acid concentration was an independent risk factor for poor blood pressure control in hypertension subjects. Many studies showed that higher uric acid levels are associated with the development of various inflammatory conditions, such as type 2 diabetes mellitus, obesity, and metabolic syndrome [22–24]. Some studies [25–27] showed that the Uric acid to High-density lipoprotein cholesterol ratio (UHR) was significantly higher in hepatic steatosis, metabolic syndrome and diabetes mellitus.

This study was the first to analyze whether uric acid was a risk factor for DKA in AP patients with diabetes mellitus, and the results showed that the blood uric acid concentration in DKA patients is higher than that in non-DKA patients. The reason may be that uric acid, as a pro-inflammatory factor, may aggravate the inflammatory response in patients with pancreatitis, thus affecting the secretion of insulin by islet cells. Insufficient insulin production leaded to elevated blood glucose, which further leaded to DKA.

As only 1 patient in both groups had severe pancreatitis, although the DKA group had a higher rate of severe pancreatitis than the non-DKA group, the difference was no statistically significant. If the study had a larger sample size, the results might be different.

This study has some limitations. First, we only included AP patients with diabetes. Although some AP patients had no diabetes in the past, they may also be complicated with hyperglycemia during hospitalization, and these patients were not included. Secondly, all the patients we enrolled had type 2 diabetes and none had type 1 diabetes. Last, in this study, there were only 25 patients, the sample size was too small, and it was a single center study. Therefore, it is hoped that further studies will be conducted in the future and more patients will be included.

In conclusion, the results of this study are of great clinical significance. The incidence of DKA in AP patients with diabetes may be much higher, so blood glucose should be closely monitored during hospitalization. Age, BMI, worse glycemic control and uric acid concentration may be predictors of DKA in AP patients with diabetes.

## Electronic supplementary material

Below is the link to the electronic supplementary material.

Supplementary Material 1

## Data Availability

The datasets in the current study are available from the corresponding author. We also provide it as supplementary information file.
